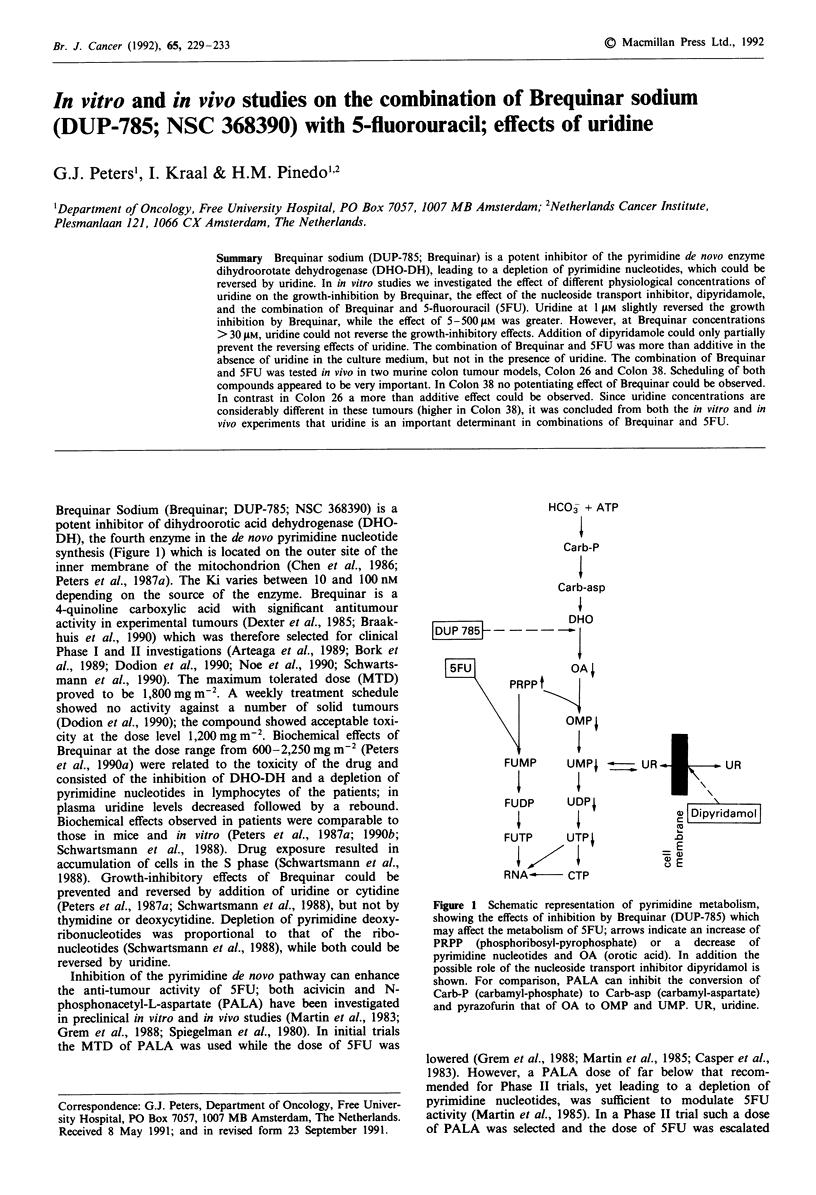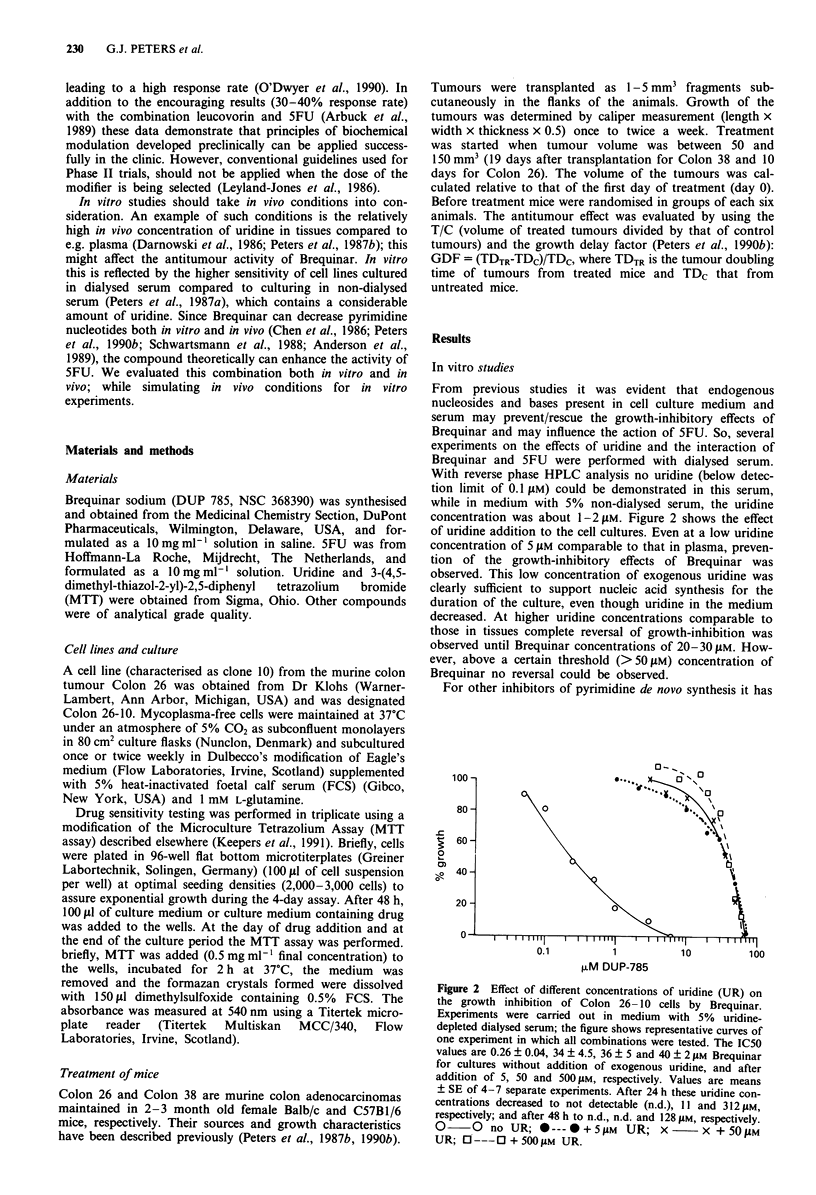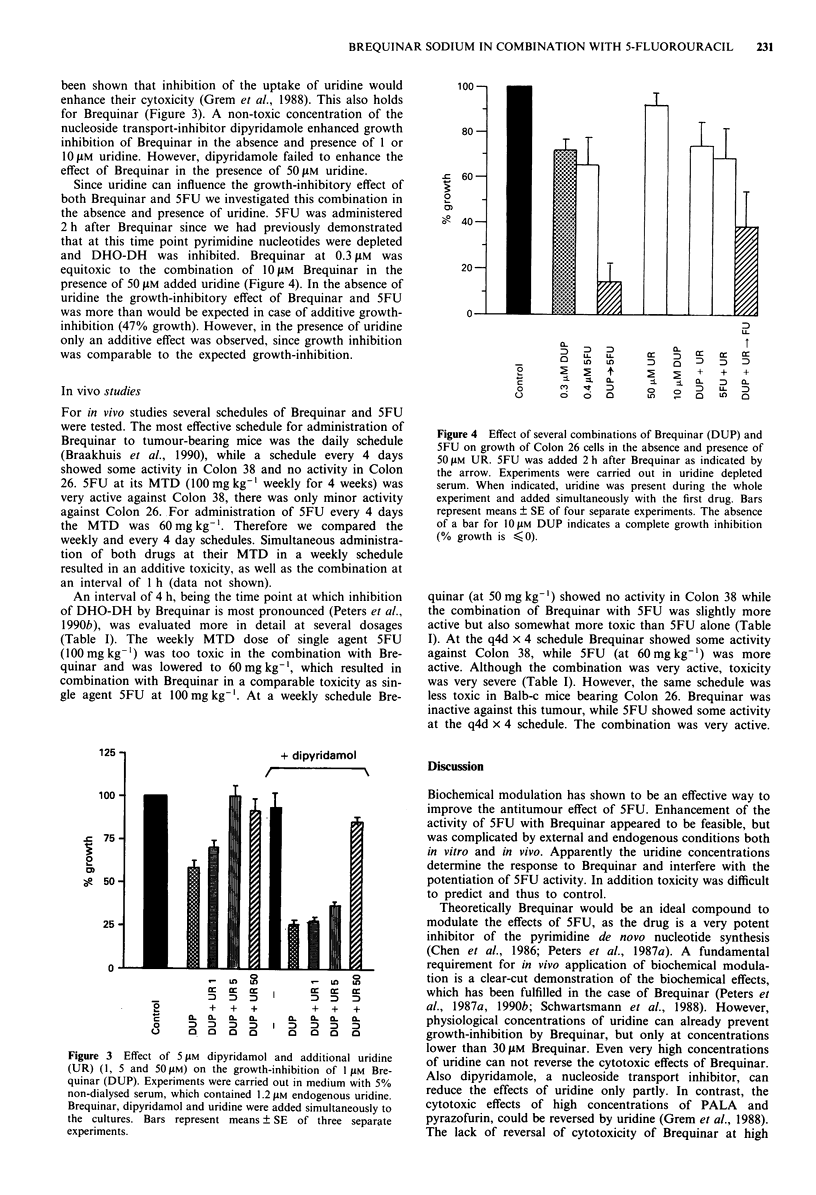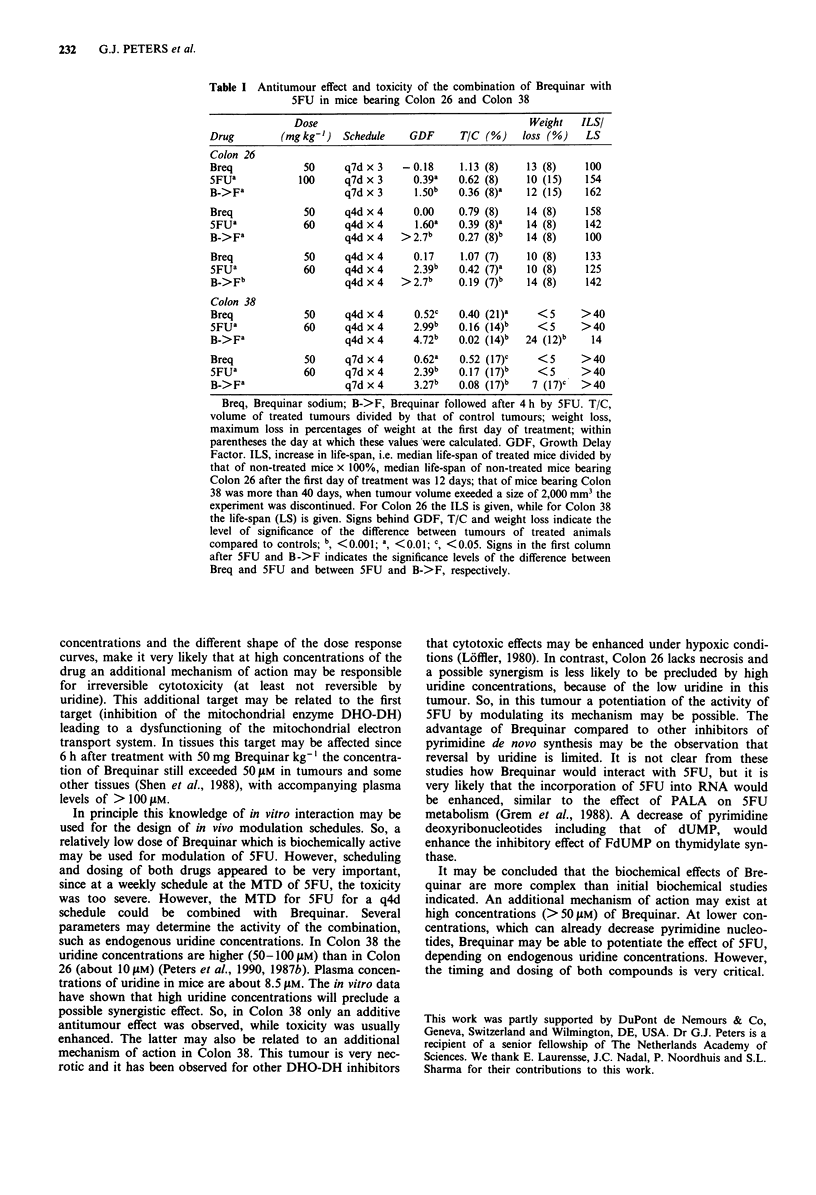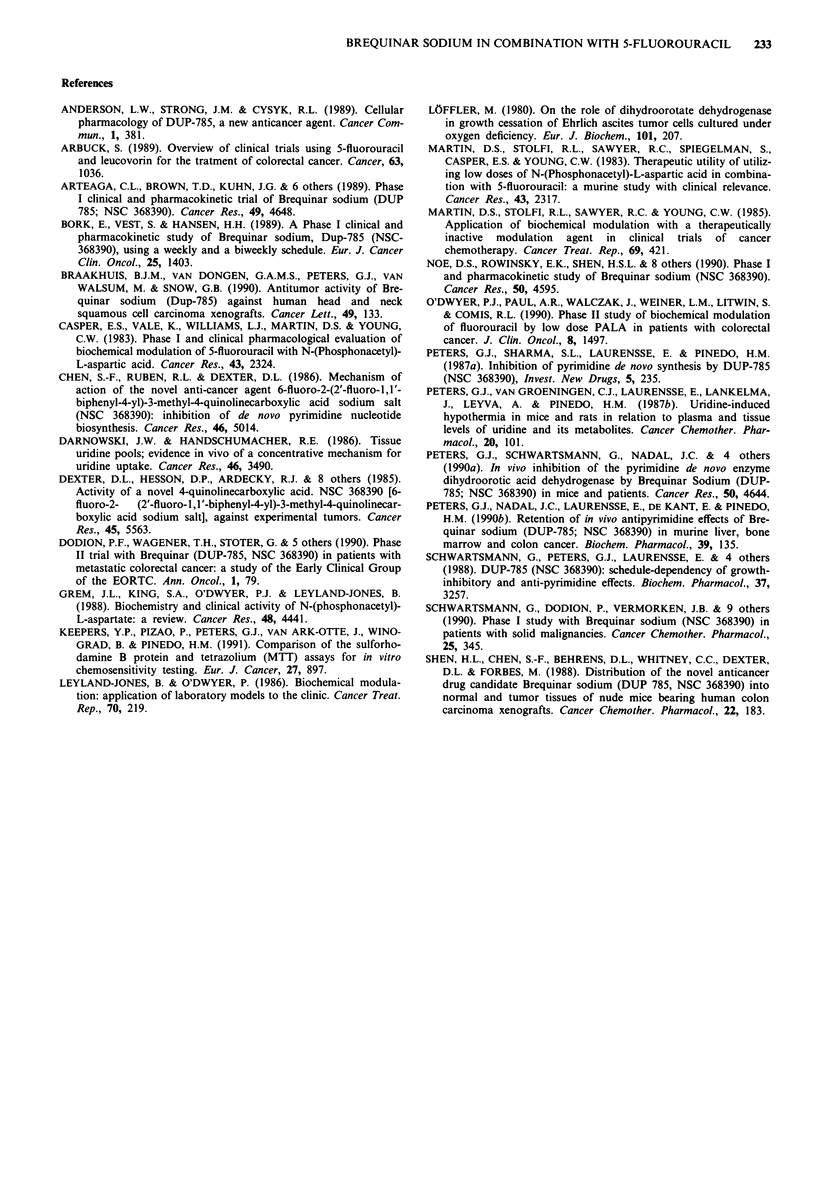# In vitro and in vivo studies on the combination of Brequinar sodium (DUP-785; NSC 368390) with 5-fluorouracil; effects of uridine.

**DOI:** 10.1038/bjc.1992.46

**Published:** 1992-02

**Authors:** G. J. Peters, I. Kraal, H. M. Pinedo

**Affiliations:** Department of Oncology, Free University Hospital, Amsterdam, The Netherlands.

## Abstract

Brequinar sodium (DUP-785; Brequinar) is a potent inhibitor of the pyrimidine de novo enzyme dihydroorotate dehydrogenase (DHO-DH), leading to a depletion of pyrimidine nucleotides, which could be reversed by uridine. In in vitro studies we investigated the effect of different physiological concentrations of uridine on the growth-inhibition by Brequinar, the effect of the nucleoside transport inhibitor, dipyridamole, and the combination of Brequinar and 5-fluorouracil (5FU). Uridine at 1 microM slightly reversed the growth inhibition by Brequinar, while the effect of 5-500 microM was greater. However, at Brequinar concentrations greater than 30 microM, uridine could not reverse the growth-inhibitory effects. Addition of dipyridamole could only partially prevent the reversing effects of uridine. The combination of Brequinar and 5FU was more than additive in the absence of uridine in the culture medium, but not in the presence of uridine. The combination of Brequinar and 5FU was tested in vivo in two murine colon tumour models, Colon 26 and Colon 38. Scheduling of both compounds appeared to be very important. In Colon 38 no potentiating effect of Brequinar could be observed. In contrast in Colon 26 a more than additive effect could be observed. Since uridine concentrations are considerably different in these tumours (higher in Colon 38), it was concluded from both the in vitro and in vivo experiments that uridine is an important determinant in combinations of Brequinar and 5FU.


*Br. J. Cancer* (1992), **65**, 229–233 © Macmillan Press Ltd., 1992

### *In vitro* and *in vivo* studies on the combination of Brequinar sodium (DUP-785; NSC 368390) with 5-fluorouracil; effects of uridine

G.J. Peters[1], I. Kraal & H.M. Pinedo[1,2]

[1]*Department of Oncology, Free University Hospital, PO Box 7057, 1007 MB Amsterdam;* [2]*Netherlands Cancer Institute, Plesmanlaan 121, 1066 CX Amsterdam, The Netherlands.*

**Summary** Brequinar sodium (DUP-785; Brequinar) is a potent inhibitor of the pyrimidine *de novo* enzyme dihydroorotate dehydrogenase (DHO-DH), leading to a depletion of pyrimidine nucleotides, which could be reversed by uridine. In *in vitro* studies we investigated the effect of different physiological concentrations of uridine on the growth-inhibition by Brequinar, the effect of the nucleoside transport inhibitor, dipyridamole, and the combination of Brequinar and 5-fluorouracil (5FU). Uridine at 1 μM slightly reversed the growth inhibition by Brequinar, while the effect of 5–500 μM was greater. However, at Brequinar concentrations >30 μM, uridine could not reverse the growth-inhibitory effects. Addition of dipyridamole could only partially prevent the reversing effects of uridine. The combination of Brequinar and 5FU was more than additive in the absence of uridine in the culture medium, but not in the presence of uridine. The combination of Brequinar and 5FU was tested *in vivo* in two murine colon tumour models, Colon 26 and Colon 38. Scheduling of both compounds appeared to be very important. In Colon 38 no potentiating effect of Brequinar could be observed. In contrast in Colon 26 a more than additive effect could be observed. Since uridine concentrations are considerably different in these tumours (higher in Colon 38), it was concluded from both the *in vitro* and *in vivo* experiments that uridine is an important determinant in combinations of Brequinar and 5FU.

Brequinar Sodium (Brequinar; DUP-785; NSC 368390) is a potent inhibitor of dihydroorotic acid dehydrogenase (DHO-DH), the fourth enzyme in the *de novo* pyrimidine nucleotide synthesis (Figure 1) which is located on the outer site of the inner membrane of the mitochondrion (Chen *et al.*, 1986; Peters *et al.*, 1987*a*). The Ki varies between 10 and 100 nM depending on the source of the enzyme. Brequinar is a 4-quinoline carboxylic acid with significant antitumour activity in experimental tumours (Dexter *et al.*, 1985; Braakhuis *et al.*, 1990) which was therefore selected for clinical Phase I and II investigations (Arteaga *et al.*, 1989; Bork *et al.*, 1989; Dodion *et al.*, 1990; Noe *et al.*, 1990; Schwartsmann *et al.*, 1990). The maximum tolerated dose (MTD) proved to be 1,800 mg m$^{-2}$. A weekly treatment schedule showed no activity against a number of solid tumours (Dodion *et al.*, 1990); the compound showed acceptable toxicity at the dose level 1,200 mg m$^{-2}$. Biochemical effects of Brequinar at the dose range from 600–2,250 mg m$^{-2}$ (Peters *et al.*, 1990*a*) were related to the toxicity of the drug and consisted of the inhibition of DHO-DH and a depletion of pyrimidine nucleotides in lymphocytes of the patients; in plasma uridine levels decreased followed by a rebound. Biochemical effects observed in patients were comparable to those in mice and *in vitro* (Peters *et al.*, 1987*a*; 1990*b*; Schwartsmann *et al.*, 1988). Drug exposure resulted in accumulation of cells in the S phase (Schwartsmann *et al.*, 1988). Growth-inhibitory effects of Brequinar could be prevented and reversed by addition of uridine or cytidine (Peters *et al.*, 1987*a*; Schwartsmann *et al.*, 1988), but not by thymidine or deoxycytidine. Depletion of pyrimidine deoxyribonucleotides was proportional to that of the ribonucleotides (Schwartsmann *et al.*, 1988), while both could be reversed by uridine.

Inhibition of the pyrimidine *de novo* pathway can enhance the anti-tumour activity of 5FU; both acivicin and N-phosphonacetyl-L-aspartate (PALA) have been investigated in preclinical *in vitro* and *in vivo* studies (Martin *et al.*, 1983; Grem *et al.*, 1988; Spiegelman *et al.*, 1980). In initial trials the MTD of PALA was used while the dose of 5FU was lowered (Grem *et al.*, 1988; Martin *et al.*, 1985; Casper *et al.*, 1983). However, a PALA dose of far below that recommended for Phase II trials, yet leading to a depletion of pyrimidine nucleotides, was sufficient to modulate 5FU activity (Martin *et al.*, 1985). In a Phase II trial such a dose of PALA was selected and the dose of 5FU was escalated

**Figure 1** Schematic representation of pyrimidine metabolism, showing the effects of inhibition by Brequinar (DUP-785) which may affect the metabolism of 5FU; arrows indicate an increase of PRPP (phosphoribosyl-pyrophosphate) or a decrease of pyrimidine nucleotides and OA (orotic acid). In addition the possible role of the nucleoside transport inhibitor dipyridamol is shown. For comparison, PALA can inhibit the conversion of Carb-P (carbamyl-phosphate) to Carb-asp (carbamyl-aspartate) and pyrazofurin that of OA to OMP and UMP. UR, uridine.

Correspondence: G.J. Peters, Department of Oncology, Free University Hospital, PO Box 7057, 1007 MB Amsterdam, The Netherlands.
Received 8 May 1991; and in revised form 23 September 1991.

leading to a high response rate (O'Dwyer *et al.*, 1990). In addition to the encouraging results (30–40% response rate) with the combination leucovorin and 5FU (Arbuck *et al.*, 1989) these data demonstrate that principles of biochemical modulation developed preclinically can be applied successfully in the clinic. However, conventional guidelines used for Phase II trials, should not be applied when the dose of the modifier is being selected (Leyland-Jones *et al.*, 1986).

*In vitro* studies should take *in vivo* conditions into consideration. An example of such conditions is the relatively high *in vivo* concentration of uridine in tissues compared to e.g. plasma (Darnowski *et al.*, 1986; Peters *et al.*, 1987*b*); this might affect the antitumour activity of Brequinar. *In vitro* this is reflected by the higher sensitivity of cell lines cultured in dialysed serum compared to culturing in non-dialysed serum (Peters *et al.*, 1987*a*), which contains a considerable amount of uridine. Since Brequinar can decrease pyrimidine nucleotides both *in vitro* and *in vivo* (Chen *et al.*, 1986; Peters *et al.*, 1990*b*; Schwartsmann *et al.*, 1988; Anderson *et al.*, 1989), the compound theoretically can enhance the activity of 5FU. We evaluated this combination both *in vitro* and *in vivo*; while simulating *in vivo* conditions for *in vitro* experiments.

### Materials and methods

### *Materials*

Brequinar sodium (DUP 785, NSC 368390) was synthesised and obtained from the Medicinal Chemistry Section, DuPont Pharmaceuticals, Wilmington, Delaware, USA, and formulated as a 10 mg $ml^{-1}$ solution in saline. 5FU was from Hoffmann-La Roche, Mijdrecht, The Netherlands, and formulated as a 10 mg $ml^{-1}$ solution. Uridine and 3-(4,5-dimethyl-thiazol-2-yl)-2,5-diphenyl tetrazolium bromide (MTT) were obtained from Sigma, Ohio. Other compounds were of analytical grade quality.

### *Cell lines and culture*

A cell line (characterised as clone 10) from the murine colon tumour Colon 26 was obtained from Dr Klohs (Warner-Lambert, Ann Arbor, Michigan, USA) and was designated Colon 26-10. Mycoplasma-free cells were maintained at 37°C under an atmosphere of 5% $CO_2$ as subconfluent monolayers in 80 $cm^2$ culture flasks (Nunclon, Denmark) and subcultured once or twice weekly in Dulbecco's modification of Eagle's medium (Flow Laboratories, Irvine, Scotland) supplemented with 5% heat-inactivated foetal calf serum (FCS) (Gibco, New York, USA) and 1 mM L-glutamine.

Drug sensitivity testing was performed in triplicate using a modification of the Microculture Tetrazolium Assay (MTT assay) described elsewhere (Keepers *et al.*, 1991). Briefly, cells were plated in 96-well flat bottom microtiterplates (Greiner Labortechnik, Solingen, Germany) (100 µl of cell suspension per well) at optimal seeding densities (2,000–3,000 cells) to assure exponential growth during the 4-day assay. After 48 h, 100 µl of culture medium or culture medium containing drug was added to the wells. At the day of drug addition and at the end of the culture period the MTT assay was performed. briefly, MTT was added (0.5 mg $ml^{-1}$ final concentration) to the wells, incubated for 2 h at 37°C, the medium was removed and the formazan crystals formed were dissolved with 150 µl dimethylsulfoxide containing 0.5% FCS. The absorbance was measured at 540 nm using a Titertek microplate reader (Titertek Multiskan MCC/340, Flow Laboratories, Irvine, Scotland).

### *Treatment of mice*

Colon 26 and Colon 38 are murine colon adenocarcinomas maintained in 2–3 month old female Balb/c and C57Bl/6 mice, respectively. Their sources and growth characteristics have been described previously (Peters *et al.*, 1987*b*, 1990*b*). Tumours were transplanted as 1–5 $mm^3$ fragments subcutaneously in the flanks of the animals. Growth of the tumours was determined by caliper measurement (length × width × thickness × 0.5) once to twice a week. Treatment was started when tumour volume was between 50 and 150 $mm^3$ (19 days after transplantation for Colon 38 and 10 days for Colon 26). The volume of the tumours was calculated relative to that of the first day of treatment (day 0). Before treatment mice were randomised in groups of each six animals. The antitumour effect was evaluated by using the T/C (volume of treated tumours divided by that of control tumours) and the growth delay factor (Peters *et al.*, 1990*b*): $GDF = (TD_{TR}-TD_C)/TD_C$, where $TD_{TR}$ is the tumour doubling time of tumours from treated mice and $TD_C$ that from untreated mice.

### Results

### In vitro *studies*

From previous studies it was evident that endogenous nucleosides and bases present in cell culture medium and serum may prevent/rescue the growth-inhibitory effects of Brequinar and may influence the action of 5FU. So, several experiments on the effects of uridine and the interaction of Brequinar and 5FU were performed with dialysed serum. With reverse phase HPLC analysis no uridine (below detection limit of 0.1 µM) could be demonstrated in this serum, while in medium with 5% non-dialysed serum, the uridine concentration was about 1–2 µM. Figure 2 shows the effect of uridine addition to the cell cultures. Even at a low uridine concentration of 5 µM comparable to that in plasma, prevention of the growth-inhibitory effects of Brequinar was observed. This low concentration of exogenous uridine was clearly sufficient to support nucleic acid synthesis for the duration of the culture, even though uridine in the medium decreased. At higher uridine concentrations comparable to those in tissues complete reversal of growth-inhibition was observed until Brequinar concentrations of 20–30 µM. However, above a certain threshold (>50 µM) concentration of Brequinar no reversal could be observed.

For other inhibitors of pyrimidine *de novo* synthesis it has

**Figure 2** Effect of different concentrations of uridine (UR) on the growth inhibition of Colon 26-10 cells by Brequinar. Experiments were carried out in medium with 5% uridine-depleted dialysed serum; the figure shows representative curves of one experiment in which all combinations were tested. The IC50 values are 0.26 ± 0.04, 34 ± 4.5, 36 ± 5 and 40 ± 2 µM Brequinar for cultures without addition of exogenous uridine, and after addition of 5, 50 and 500 µM, respectively. Values are means ± SE of 4–7 separate experiments. After 24 h these uridine concentrations decreased to not detectable (n.d.), 11 and 312 µM, respectively; and after 48 h to n.d., n.d. and 128 µM, respectively. O——O no UR; ●---● + 5 µM UR; ×——× + 50 µM UR; □---□ + 500 µM UR.

been shown that inhibition of the uptake of uridine would enhance their cytoxicity (Grem *et al.*, 1988). This also holds for Brequinar (Figure 3). A non-toxic concentration of the nucleoside transport-inhibitor dipyridamole enhanced growth inhibition of Brequinar in the absence and presence of 1 or 10 μM uridine. However, dipyridamole failed to enhance the effect of Brequinar in the presence of 50 μM uridine.

Since uridine can influence the growth-inhibitory effect of both Brequinar and 5FU we investigated this combination in the absence and presence of uridine. 5FU was administered 2 h after Brequinar since we had previously demonstrated that at this time point pyrimidine nucleotides were depleted and DHO-DH was inhibited. Brequinar at 0.3 μM was equitoxic to the combination of 10 μM Brequinar in the presence of 50 μM added uridine (Figure 4). In the absence of uridine the growth-inhibitory effect of Brequinar and 5FU was more than would be expected in case of additive growth-inhibition (47% growth). However, in the presence of uridine only an additive effect was observed, since growth inhibition was comparable to the expected growth-inhibition.

**Figure 4** Effect of several combinations of Brequinar (DUP) and 5FU on growth of Colon 26 cells in the absence and presence of 50 μM UR. 5FU was added 2 h after Brequinar as indicated by the arrow. Experiments were carried out in uridine depleted serum. When indicated, uridine was present during the whole experiment and added simultaneously with the first drug. Bars represent means ± SE of four separate experiments. The absence of a bar for 10 μM DUP indicates a complete growth inhibition (% growth is ⩽0).

### In vivo *studies*

For *in vivo* studies several schedules of Brequinar and 5FU were tested. The most effective schedule for administration of Brequinar to tumour-bearing mice was the daily schedule (Braakhuis *et al.*, 1990), while a schedule every 4 days showed some activity in Colon 38 and no activity in Colon 26. 5FU at its MTD (100 mg kg$^{-1}$ weekly for 4 weeks) was very active against Colon 38, there was only minor activity against Colon 26. For administration of 5FU every 4 days the MTD was 60 mg kg$^{-1}$. Therefore we compared the weekly and every 4 day schedules. Simultaneous administration of both drugs at their MTD in a weekly schedule resulted in an additive toxicity, as well as the combination at an interval of 1 h (data not shown).

An interval of 4 h, being the time point at which inhibition of DHO-DH by Brequinar is most pronounced (Peters *et al.*, 1990*b*), was evaluated more in detail at several dosages (Table I). The weekly MTD dose of single agent 5FU (100 mg kg$^{-1}$) was too toxic in the combination with Brequinar and was lowered to 60 mg kg$^{-1}$, which resulted in combination with Brequinar in a comparable toxicity as single agent 5FU at 100 mg kg$^{-1}$. At a weekly schedule Brequinar (at 50 mg kg$^{-1}$) showed no activity in Colon 38 while the combination of Brequinar with 5FU was slightly more active but also somewhat more toxic than 5FU alone (Table I). At the q4d × 4 schedule Brequinar showed some activity against Colon 38, while 5FU (at 60 mg kg$^{-1}$) was more active. Although the combination was very active, toxicity was very severe (Table I). However, the same schedule was less toxic in Balb-c mice bearing Colon 26. Brequinar was inactive against this tumour, while 5FU showed some activity at the q4d × 4 schedule. The combination was very active.

**Figure 3** Effect of 5 μM dipyridamol and additional uridine (UR) (1, 5 and 50 μM) on the growth-inhibition of 1 μM Brequinar (DUP). Experiments were carried out in medium with 5% non-dialysed serum, which contained 1.2 μM endogenous uridine. Brequinar, dipyridamol and uridine were added simultaneously to the cultures. Bars represent means ± SE of three separate experiments.

### Discussion

Biochemical modulation has shown to be an effective way to improve the antitumour effect of 5FU. Enhancement of the activity of 5FU with Brequinar appeared to be feasible, but was complicated by external and endogenous conditions both *in vitro* and *in vivo*. Apparently the uridine concentrations determine the response to Brequinar and interfere with the potentiation of 5FU activity. In addition toxicity was difficult to predict and thus to control.

Theoretically Brequinar would be an ideal compound to modulate the effects of 5FU, as the drug is a very potent inhibitor of the pyrimidine *de novo* nucleotide synthesis (Chen *et al.*, 1986; Peters *et al.*, 1987*a*). A fundamental requirement for *in vivo* application of biochemical modulation is a clear-cut demonstration of the biochemical effects, which has been fulfilled in the case of Brequinar (Peters *et al.*, 1987*a*, 1990*b*; Schwartsmann *et al.*, 1988). However, physiological concentrations of uridine can already prevent growth-inhibition by Brequinar, but only at concentrations lower than 30 μM Brequinar. Even very high concentrations of uridine can not reverse the cytotoxic effects of Brequinar. Also dipyridamole, a nucleoside transport inhibitor, can reduce the effects of uridine only partly. In contrast, the cytotoxic effects of high concentrations of PALA and pyrazofurin, could be reversed by uridine (Grem *et al.*, 1988). The lack of reversal of cytotoxicity of Brequinar at high

**Table I** Antitumour effect and toxicity of the combination of Brequinar with 5FU in mice bearing Colon 26 and Colon 38

| *Drug* | *Dose (mg kg$^{-1}$)* | *Schedule* | *GDF* | *T/C (%)* | *Weight loss (%)* | *ILS/ LS* |
|---|---|---|---|---|---|---|
| *Colon 26* | | | | | | |
| Breq | 50 | q7d × 3 | −0.18 | 1.13 (8) | 13 (8) | 100 |
| 5FU[a] | 100 | q7d × 3 | 0.39[a] | 0.62 (8) | 10 (15) | 154 |
| B->F[a] | | q7d × 3 | 1.50[b] | 0.36 (8)[a] | 12 (15) | 162 |
| Breq | 50 | q4d × 4 | 0.00 | 0.79 (8) | 14 (8) | 158 |
| 5FU[a] | 60 | q4d × 4 | 1.60[a] | 0.39 (8)[a] | 14 (8) | 142 |
| B->F[a] | | q4d × 4 | >2.7[b] | 0.27 (8)[b] | 14 (8) | 100 |
| Breq | 50 | q4d × 4 | 0.17 | 1.07 (7) | 10 (8) | 133 |
| 5FU[a] | 60 | q4d × 4 | 2.39[b] | 0.42 (7)[a] | 10 (8) | 125 |
| B->F[b] | | q4d × 4 | >2.7[b] | 0.19 (7)[b] | 14 (8) | 142 |
| *Colon 38* | | | | | | |
| Breq | 50 | q4d × 4 | 0.52[c] | 0.40 (21)[a] | <5 | >40 |
| 5FU[a] | 60 | q4d × 4 | 2.99[b] | 0.16 (14)[b] | <5 | >40 |
| B->F[a] | | q4d × 4 | 4.72[b] | 0.02 (14)[b] | 24 (12)[b] | 14 |
| Breq | 50 | q7d × 4 | 0.62[a] | 0.52 (17)[c] | <5 | >40 |
| 5FU[a] | 60 | q7d × 4 | 2.39[b] | 0.17 (17)[b] | <5 | >40 |
| B->F[a] | | q7d × 4 | 3.27[b] | 0.08 (17)[b] | 7 (17)[c] | >40 |

Breq, Brequinar sodium; B->F, Brequinar followed after 4 h by 5FU. T/C, volume of treated tumours divided by that of control tumours; weight loss, maximum loss in percentages of weight at the first day of treatment; within parentheses the day at which these values were calculated. GDF, Growth Delay Factor. ILS, increase in life-span, i.e. median life-span of treated mice divided by that of non-treated mice × 100%, median life-span of non-treated mice bearing Colon 26 after the first day of treatment was 12 days; that of mice bearing Colon 38 was more than 40 days, when tumour volume exeeded a size of 2,000 mm$^3$ the experiment was discontinued. For Colon 26 the ILS is given, while for Colon 38 the life-span (LS) is given. Signs behind GDF, T/C and weight loss indicate the level of significance of the difference between tumours of treated animals compared to controls; [b], <0.001; [a], <0.01; [c], <0.05. Signs in the first column after 5FU and B->F indicates the significance levels of the difference between Breq and 5FU and between 5FU and B->F, respectively.

concentrations and the different shape of the dose response curves, make it very likely that at high concentrations of the drug an additional mechanism of action may be responsible for irreversible cytotoxicity (at least not reversible by uridine). This additional target may be related to the first target (inhibition of the mitochondrial enzyme DHO-DH) leading to a dysfunctioning of the mitochondrial electron transport system. In tissues this target may be affected since 6 h after treatment with 50 mg Brequinar kg$^{-1}$ the concentration of Brequinar still exceeded 50 μM in tumours and some other tissues (Shen *et al.*, 1988), with accompanying plasma levels of > 100 μM.

In principle this knowledge of *in vitro* interaction may be used for the design of *in vivo* modulation schedules. So, a relatively low dose of Brequinar which is biochemically active may be used for modulation of 5FU. However, scheduling and dosing of both drugs appeared to be very important, since at a weekly schedule at the MTD of 5FU, the toxicity was too severe. However, the MTD for 5FU for a q4d schedule could be combined with Brequinar. Several parameters may determine the activity of the combination, such as endogenous uridine concentrations. In Colon 38 the uridine concentrations are higher (50–100 μM) than in Colon 26 (about 10 μM) (Peters *et al.*, 1990, 1987*b*). Plasma concentrations of uridine in mice are about 8.5 μM. The *in vitro* data have shown that high uridine concentrations will preclude a possible synergistic effect. So, in Colon 38 only an additive antitumour effect was observed, while toxicity was usually enhanced. The latter may also be related to an additional mechanism of action in Colon 38. This tumour is very necrotic and it has been observed for other DHO-DH inhibitors that cytotoxic effects may be enhanced under hypoxic conditions (Löffler, 1980). In contrast, Colon 26 lacks necrosis and a possible synergism is less likely to be precluded by high uridine concentrations, because of the low uridine in this tumour. So, in this tumour a potentiation of the activity of 5FU by modulating its mechanism may be possible. The advantage of Brequinar compared to other inhibitors of pyrimidine *de novo* synthesis may be the observation that reversal by uridine is limited. It is not clear from these studies how Brequinar would interact with 5FU, but it is very likely that the incorporation of 5FU into RNA would be enhanced, similar to the effect of PALA on 5FU metabolism (Grem *et al.*, 1988). A decrease of pyrimidine deoxyribonucleotides including that of dUMP, would enhance the inhibitory effect of FdUMP on thymidylate synthase.

It may be concluded that the biochemical effects of Brequinar are more complex than initial biochemical studies indicated. An additional mechanism of action may exist at high concentrations (> 50 μM) of Brequinar. At lower concentrations, which can already decrease pyrimidine nucleotides, Brequinar may be able to potentiate the effect of 5FU, depending on endogenous uridine concentrations. However, the timing and dosing of both compounds is very critical.

This work was partly supported by DuPont de Nemours & Co, Geneva, Switzerland and Wilmington, DE, USA. Dr G.J. Peters is a recipient of a senior fellowship of The Netherlands Academy of Sciences. We thank E. Laurensse, J.C. Nadal, P. Noordhuis and S.L. Sharma for their contributions to this work.

### References

ANDERSON, L.W., STRONG, J.M. & CYSYK, R.L. (1989). Cellular pharmacology of DUP-785, a new anticancer agent. *Cancer Commun.*, **1**, 381.

ARBUCK, S. (1989). Overview of clinical trials using 5-fluorouracil and leucovorin for the tratment of colorectal cancer. *Cancer*, **63**, 1036.

ARTEAGA, C.L., BROWN, T.D., KUHN, J.G. & 6 others (1989). Phase I clinical and pharmacokinetic trial of Brequinar sodium (DUP 785; NSC 368390). *Cancer Res.*, **49**, 4648.

BORK, E., VEST, S. & HANSEN, H.H. (1989). A Phase I clinical and pharmacokinetic study of Brequinar sodium, Dup-785 (NSC-368390), using a weekly and a biweekly schedule. *Eur. J. Cancer Clin. Oncol.*, **25**, 1403.

BRAAKHUIS, B.J.M., VAN DONGEN, G.A.M.S., PETERS, G.J., VAN WALSUM, M. & SNOW, G.B. (1990). Antitumor activity of Brequinar sodium (Dup-785) against human head and neck squamous cell carcinoma xenografts. *Cancer Lett.*, **49**, 133.

CASPER, E.S., VALE, K., WILLIAMS, L.J., MARTIN, D.S. & YOUNG, C.W. (1983). Phase I and clinical pharmacological evaluation of biochemical modulation of 5-fluorouracil with N-(Phosphonacetyl)-L-aspartic acid. *Cancer Res.*, **43**, 2324.

CHEN, S.-F., RUBEN, R.L. & DEXTER, D.L. (1986). Mechanism of action of the novel anti-cancer agent 6-fluoro-2-(2′-fluoro-1,1′-biphenyl-4-yl)-3-methyl-4-quinolinecarboxylic acid sodium salt (NSC 368390): inhibition of *de novo* pyrimidine nucleotide biosynthesis. *Cancer Res.*, **46**, 5014.

DARNOWSKI, J.W. & HANDSCHUMACHER, R.E. (1986). Tissue uridine pools; evidence in vivo of a concentrative mechanism for uridine uptake. *Cancer Res.*, **46**, 3490.

DEXTER, D.L., HESSON, D.P., ARDECKY, R.J. & 8 others (1985). Activity of a novel 4-quinolinecarboxylic acid. NSC 368390 [6-fluoro-2- (2′-fluoro-1,1′-biphenyl-4-yl)-3-methyl-4-quinolinecarboxylic acid sodium salt], against experimental tumors. *Cancer Res.*, **45**, 5563.

DODION, P.F., WAGENER, T.H., STOTER, G. & 5 others (1990). Phase II trial with Brequinar (DUP-785, NSC 368390) in patients with metastatic colorectal cancer: a study of the Early Clinical Group of the EORTC. *Ann. Oncol.*, **1**, 79.

GREM, J.L., KING, S.A., O'DWYER, P.J. & LEYLAND-JONES, B. (1988). Biochemistry and clinical activity of N-(phosphonacetyl)-L-aspartate: a review. *Cancer Res.*, **48**, 4441.

KEEPERS, Y.P., PIZAO, P., PETERS, G.J., VAN ARK-OTTE, J., WINOGRAD, B. & PINEDO, H.M. (1991). Comparison of the sulforhodamine B protein and tetrazolium (MTT) assays for *in vitro* chemosensitivity testing. *Eur. J. Cancer*, **27**, 897.

LEYLAND-JONES, B. & O'DWYER, P. (1986). Biochemical modulation: application of laboratory models to the clinic. *Cancer Treat. Rep.*, **70**, 219.

LÖFFLER, M. (1980). On the role of dihydroorotate dehydrogenase in growth cessation of Ehrlich ascites tumor cells cultured under oxygen deficiency. *Eur. J. Biochem.*, **101**, 207.

MARTIN, D.S., STOLFI, R.L., SAWYER, R.C., SPIEGELMAN, S., CASPER, E.S. & YOUNG, C.W. (1983). Therapeutic utility of utilizing low doses of N-(Phosphonacetyl)-L-aspartic acid in combination with 5-fluorouracil: a murine study with clinical relevance. *Cancer Res.*, **43**, 2317.

MARTIN, D.S., STOLFI, R.L., SAWYER, R.C. & YOUNG, C.W. (1985). Application of biochemical modulation with a therapeutically inactive modulation agent in clinical trials of cancer chemotherapy. *Cancer Treat. Rep.*, **69**, 421.

NOE, D.S., ROWINSKY, E.K., SHEN, H.S.L. & 8 others (1990). Phase I and pharmacokinetic study of Brequinar sodium (NSC 368390). *Cancer Res.*, **50**, 4595.

O'DWYER, P.J., PAUL, A.R., WALCZAK, J., WEINER, L.M., LITWIN, S. & COMIS, R.L. (1990). Phase II study of biochemical modulation of fluorouracil by low dose PALA in patients with colorectal cancer. *J. Clin. Oncol.*, **8**, 1497.

PETERS, G.J., SHARMA, S.L., LAURENSSE, E. & PINEDO, H.M. (1987*a*). Inhibition of pyrimidine *de novo* synthesis by DUP-785 (NSC 368390), *Invest. New Drugs*, **5**, 235.

PETERS, G.J., VAN GROENINGEN, C.J., LAURENSSE, E., LANKELMA, J., LEYVA, A. & PINEDO, H.M. (1987*b*). Uridine-induced hypothermia in mice and rats in relation to plasma and tissue levels of uridine and its metabolites. *Cancer Chemother. Pharmacol.*, **20**, 101.

PETERS, G.J., SCHWARTSMANN, G., NADAL, J.C. & 4 others (1990*a*). *In vivo* inhibition of the pyrimidine *de novo* enzyme dihydroorotic acid dehydrogenase by Brequinar Sodium (DUP-785; NSC 368390) in mice and patients. *Cancer Res.*, **50**, 4644.

PETERS, G.J., NADAL, J.C., LAURENSSE, E., DE KANT, E. & PINEDO, H.M. (1990*b*). Retention of *in vivo* antipyrimidine effects of Brequinar sodium (DUP-785; NSC 368390) in murine liver, bone marrow and colon cancer. *Biochem. Pharmacol.*, **39**, 135.

SCHWARTSMANN, G., PETERS, G.J., LAURENSSE, E. & 4 others (1988). DUP-785 (NSC 368390): schedule-dependency of growth-inhibitory and anti-pyrimidine effects. *Biochem. Pharmacol.*, **37**, 3257.

SCHWARTSMANN, G., DODION, P., VERMORKEN, J.B. & 9 others (1990). Phase I study with Brequinar sodium (NSC 368390) in patients with solid malignancies. *Cancer Chemother. Pharmacol.*, **25**, 345.

SHEN, H.L., CHEN, S.-F., BEHRENS, D.L., WHITNEY, C.C., DEXTER, D.L. & FORBES, M. (1988). Distribution of the novel anticancer drug candidate Brequinar sodium (DUP 785, NSC 368390) into normal and tumor tissues of nude mice bearing human colon carcinoma xenografts. *Cancer Chemother. Pharmacol.*, **22**, 183.